# Service impact of a change in HIV-1 viral load quantification assay

**DOI:** 10.7448/IAS.17.4.19677

**Published:** 2014-11-02

**Authors:** Craig Tipple, Soonita Oomeer, Olamide Dosekun, Nicola Mackie

**Affiliations:** Jefferiss Wing for Sexual Health, Imperial College Healthcare NHS Trust, London, UK

## Abstract

**Introduction:**

Due to discontinuation of the Siemens Versant HIV-1 RNA (bDNA) assay in the UK, our laboratory switched to the Roche Cobas Ampliprep/Taqman HIV-1 viral load (VL) assay (Roche) in April 2013. This assay has a lower cut-off of 20 RNA copies/mL (compared with <50 for the Siemens assay). Our laboratory demonstrated previously that a significant proportion (18%) of patients undetectable using bDNA HIV-1 RNA quantification exhibited low level viraemia (LLV) using the new assay. Local guidelines recommend that patients stable on therapy receive twice-yearly VLs. We evaluated the impact of the introduction of the new assay on our clinical service.

**Methods:**

A retrospective cohort analysis of treated patients with stable undetectable VL by bDNA (<50 copies/mL) followed by ≥ one low-level (<400 copies/mL) VL with the Roche assay. Demographic data were collected in addition to frequency of VL testing and genotypic resistance assays. Referrals to virtual clinic (VC) were recorded. Patients were identified using laboratory data and information collected from electronic patient records. Results were analyzed with SPSS v18.

**Results:**

One hundred and ninety patients were included. Demographics: 79.5% male; 60.6% homosexual; mean age of 46 years. Duration on stable treatment was 46.35 (std. dev. 38.15) months. Current treatment regimens were 43.3% PI-based; 43.3% NNRTI-based and 13.7% other. Patients were stratified into VL 20–49 copies/mL (n=109); VL 50–199 copies/mL (n=71) and VL 200–399 copies/mL (n=10). In total, there were 471 VLs measured of which 274 were additional as a result of the assay switch. This resulted in six HIV-1 genotype requests and 16 VC discussions (Table 1). Longer duration on HAART was associated with reduced frequency of VL testing. The relative risk of ongoing detectability according to drug class are: PI 1.62 (95% CI 1.18–2.21); NNRTI 0.507 (95% CI 0.30–0.85) and other 1.09 (95% CI 0.48–2.43).

**Conclusions:**

Changes in assay can result in difficulties in interpretation of patient results. The assay switch in our service had significant impact on patient and staff time and cost with an increase in patient recalls; increased frequency of VL measurement, genotypes and discussions in VC. Choice of assay is paramount to running an efficient and cost-effective clinical service.

**Table 1:** Impact of a change in HIV-1 viral load quantification assay

Roche VL (copies/mL)	Total no of VLs requested	No of VLs per patient	No of additional VLs	VC discussion	Genotypes requested
20–49	244	2.23	124	8	1
50–199	201	2.83	134	6	2
200–399	26	2.60	16	2	3
Total	471	n/a	274	16	6

Patients were divided into three groups according to the level of detectable viraemia on the first Roche-measured VL following an undetectable bDNA result.

